# Novel Vector Control Approaches: The Future for Prevention of Zika Virus Transmission?

**DOI:** 10.1371/journal.pmed.1002219

**Published:** 2017-01-17

**Authors:** Lorenz von Seidlein, Alexander S. Kekulé, Daniel Strickman

**Affiliations:** 1 Mahidol-Oxford Tropical Medicine Research Unit (MORU), Bangkok, Thailand; 2 Martin Luther University Halle-Wittenberg, Halle (Saale), Germany; 3 Bill & Melinda Gates Foundation, Seattle, Washington, United States of America

## Abstract

In a Perspective accompanying Abad-Franch and colleagues, Lorenz von Seidlein, Alexander Kekulé, and Daniel Strickman discuss the importance of developing effective strategies to minimize mosquito-borne transmission of human diseases.

The Zika virus (ZIKV) has received considerable attention over the past two years. In view of its threat to the developing fetus and the absence of specific antiviral therapy, the public health response must focus on prevention. The two major preventive strategies are vaccine development and vector control, in addition to avoidance of pregnancy where transmission occurs. Logistical challenges of vaccine distribution and the epidemiology of Zika, which has so far been characterised by sporadic outbreaks, may pose challenges in the deployment of a potential ZIKV vaccine. Routine mass ZIKV vaccinations in the absence of an outbreak will have a disadvantageous cost/benefit ratio. Reactive targeted vaccinations require time to implement; by the time an outbreak has been detected and a reactive vaccination campaign is under way, the number of cases would likely have already peaked and susceptible people infected. Individuals should have the option to protect themselves through a safe and protective ZIKV vaccine, for which there will be considerable demand. From a public health perspective, targeting the mosquito vector *Aedes aegypti* to control and prevent ZIKV outbreaks is currently much more attractive than the vaccine approach. Besides, a potential ZIKV vaccine would only target that single virus. The recent ZIKV outbreak in many states of Brazil occurred between two other vector-borne disease outbreaks, dengue and chikungunya [1]. In contrast, vector control has the potential to prevent several mosquito-borne infections. In this issue of *PLOS Medicine*, Fernando Abad-Franch and colleagues describe a new method to curb the *Ae*. *aegypti* population in a city in Brazil [17].

There is considerable enthusiasm for novel vector control approaches to prevent not only ZIKV infections but a range of vector-borne infections transmitted by *Ae*. *aegypti*. These mosquitoes typically bite during the day, with a clear preference for indoor activity. Because of the day-biting characteristic, insecticide-treated bed nets (ITNs) used widely for malaria control are assumed to be ineffective, although the presence of insecticide-treated material in a home may have a beneficial effect [2]. Outdoor fogging with insecticides looks dramatic and hence provides visible evidence that the government is “doing something” but requires considerable resources, provides only short-lasting benefits, and has been severely compromised by the spread of vectors resistant to the available insecticides [3]. Fogging can also affect nontarget organisms such as honeybees, moths, and butterflies—all of which can be harmed by commonly used fogging agents such as malathion and permethrin. Among the newer ideas to minimise mosquito-borne transmission of human disease has been the release of mosquitoes infected with a strain of *Wolbachia* originally isolated from *Drosophila* flies. *Ae*. *aegypti* is one of the few mosquitoes not naturally infected with its own *Wolbachia* strain, and infection with the *Drosophila* strain tends to block transmission of dengue [4], chikungunya [5], and Zika [6] viruses. This technique is currently being tested in definitive trials against dengue in Southeast Asia and in implementation trials against ZIKV in Colombia and Brazil. Another approach uses *Wolbachia* to prevent reproduction in populations of mosquitoes by introducing a strain of the bacteria into males that results in sterile eggs from all matings [6]. Very similar to the use of *Wolbachia*-infected males to prevent fertilization, a second innovative approach to vector control is the release of large numbers of sterile males. Using the sterile insect technique has contributed to the absence of agricultural pests such as the New World screwworm, *Cochliomyia hominivorax*, from North and Central America for 30 years until their return to the Florida keys earlier this year [7] and the elimination of *Glossina austeni* tsetse flies from Unguja Island, Zanzibar [8]. This technique could hold some promise against *Ae*. *aegypti* and similar vectors if wild population densities can be reduced prior to the release of the sterile males [9]. Thirdly, mosquitoes engineered to carry a lethal gene RIDL (“Release of Insects carrying a Dominant Lethal”), in which the lethal gene is repressed by tetracycline during mass rearing of mosquitoes [10]. The offspring of genetically transformed males released in the field die because of the absence of tetracycline in environmental waters. Field trials in the Cayman Islands and Brazil with such a self-limiting *Ae*. *aegypti* (designated strain OX513A) had a large impact (reduction of populations by over 95%) on wild populations [11,12]. Ironically, the release of OX513A Aedes preceding the ZIKV outbreak for the control of dengue led to conspiracy theories suggesting that the release of genetically modified mosquitoes was in some way causally related to the subsequent ZIKV outbreak [13].

In this issue of *PLOS Medicine*, Abad-Franch and colleagues describe an innovative vector control approach based on using pyriproxyfen, a very powerful synthetic analogue of mosquito juvenile hormone, to prevent ZIKV and other infections transmitted by *Ae*. *aegypti* mosquitoes. This approach has been used for decades with a different chemical called methoprene [14]. The material is nontoxic to mammals, including humans, because of the absence of the necessary endocrine pathway [15,16]. The innovative aspect of pyriproxyfen is that it can be carried in effective dosages by the mosquito itself to the larval site. Successful distribution by female mosquitoes overcomes the challenge of finding and treating every container and accumulation of water that might produce vectors. Following a smaller study in the Tancredo Neves neighbourhood of Manaus [17], the investigators report on the effectiveness of this technique in an entire city of 60,000 inhabitants, Manacapuro, which like Manaus is in the Brazilian state of Amazonas. They distributed 1,000 dissemination stations consisting of plastic cups lined with black cloth that had been treated with a powdered formulation of pyriproxyfen. Females picked up particles of the formulation when they were attracted to the cups as oviposition sites. The investigators hypothesized that enough adult mosquitoes return to larval sites after landing in the dissemination stations to make an overall impact on the mosquito populations. Despite many challenges, the authors showed that their approach was effective. Using a network of sentinel breeding sites, the investigators found that the juvenile populations of three common container-inhabiting species, including *Ae*. *aegypti*, collapsed in the months following the distribution of dissemination stations. More work in multiple sites, and with longer follow-up, will be needed to strengthen these findings and establish the longer-term effectiveness of this approach in different settings.

*Ae*. *aegypti* has been a key vector of important pathogens like yellow fever and dengue for centuries. Some programs to control the mosquitoes have been dramatically successful, such as the vector control during the construction of the Panama Canal and the complete elimination of the species from Brazil in the 1930s. The more recent uncontrolled growth of urban areas and encroachment into rural environments compounded by improvements in transportation have made control of *Ae*. *aegypti* very difficult. The rapid expansion of Zika virus and public concern with its effects on fetal development has brought more attention to the need to have efficient methods to control *Ae*. *aegypti* and other disease vectors. The right solution is likely to be a combination of eliminating as many of those breeding containers as possible and the application of both traditional and new vector control interventions.
